# Down-regulation of HIF-1α inhibits the proliferation, migration, and invasion of gastric cancer by inhibiting PI3K/AKT pathway and VEGF expression

**DOI:** 10.1042/BSR20180741

**Published:** 2018-12-21

**Authors:** Jingjing Zhang, Jun Xu, Yonghong Dong, Bo Huang

**Affiliations:** 1 The Department of General Surgery, Shanxi Provincial People’s Hospital, Taiyuan City, Shanxi Province 030012, China; 2 The Department of General Surgery, 2nd Hospital attached to Shanxi Medical University, Taiyuan City, Shanxi Province 030001, China

**Keywords:** gastric cancer, HIF-1α, PI3K/AKT pathway

## Abstract

In view of the high incidence of gastric cancer and the functions of hypoxia-inducible factor 1α (HIF-1α), our study aimed to investigate the functionality of HIF-1α in gastric cancer, and to explore the diagnostic and prognostic values of HIF-1α for this disease. Expression of HIF-1α in tumor tissues and adjacent healthy tissues as well as serum collected from both gastric cancer patients and normal healthy controls was detected by qRT-PCR. Survival analysis was performed using Kaplan–Meier method. HIF-1α siRNA silencing cell lines were established. Effects of HIF-1α siRNA silencing as well as PI3K activator sc3036 on proliferation, migration, and invasion of gastric cancer cells were detected by Cell counting kit (CCK-8) assay, and Transwell migration and invasion assay. Effects of HIF-1α siRNA silencing on AKT and VEGF were detected by Western blot. Expression of HIF-1α was significantly down-regulated in tumor tissues than in adjacent healthy tissues in most gastric cancer patients. Serum levels of HIF-1α were also higher in gastric cancer patients than in normal healthy people. Serum HIF-1α showed promising diagnostic and prognostic values for gastric cancer. HIF-1α siRNA silencing inhibited the proliferation, migration, and invasion of gastric cancer cells, while PI3K activator sc3036 treatment reduced those inhibitory effects. Down-regulation of HIF-1α can inhibit the proliferation, migration, and invasion of gastric cancer possibly by inhibiting PI3K/AKT pathway and VEGF expression.

## Background

As a type of malignancy that develops from the lining of the stomach, gastric cancer seriously affects the health and life of human [1]. With the changes in people’s lifestyle and diet structure, gastric cancer has become one of the most common malignancies worldwide. Especially in China, incidence rate of gastric is just lower than that of lung cancer and breast cancer. Gastric cancer ranks third amongst all types of malignancies [2]. With the improvement in the prevention and treatment of this disease, incidence of gastric cancer has dropped significantly in some regions during the past several decades [3]. However, gastric cancer is still one of the most common causes of cancer-related deaths because most patients were diagnosed in their advanced stages, which are not appropriate for radical resection [3]. At present, early diagnosis and treatment is still the key in improving treatment outcomes of this disease.

As hypoxia-inducible factor 1α, HIF-1α has been proved to be involved in the development and progression of various types of human cancers [4,5]. Inhibition of HIF-α expression delayed the progression of certain types of cancers such as colon cancer and ovarian cancer [6,7], indicating that the inhibition of HIF-1α expression may serve as a potential therapeutic target for different types of malignancies. HIF-1α also has been proved to participate in the pathogenesis of gastric cancer through interactions with various pathways [8,9]. However, prognostic and diagnostic values of HIF-1α for gastric cancer have not been systematically studied.

In the present study, expression of HIF-1α in tumor tissues and adjacent healthy tissues as well as serum collected from both gastric cancer patients and normal healthy people was detected. Diagnostic and prognostic values of HIF-1α for gastric cancer were analyzed, and the interactions between HIF-1α, VEGF, and PI3K/AKT pathways were explored.

## Materials and methods

### Patients

A total of 58 gastric cancer patients were enrolled from July 2009 to July 2012 in Shanxi Provincial People’s Hospital. All patients were confirmed with gastric cancer by imaging examinations (CT, MRI, and so on) and pathological examinations. Those patients included 25 males and 23 females, and age ranged from 25 to 71 years, with a mean age of 44 ± 11.2 years. Based on disease conditions, all patients received surgical resection. Tumor tissues and healthy tissues within 5 cm around the tumor were collected during the surgical operation. All normal tissues were confirmed by pathological assay. At the same time, 39 healthy people were also included to serve as control group. Control group included 17 males and 22 females, and age ranged from 23 to 72 years, with a mean age of 43 ± 13.1 years. All participants signed informed consent. The present study was approved by the Ethics Committee of Shanxi Provincial People’s Hospital. No significant differences in age and gender were found between patient group and control group. All patients were followed up for 5 years.

### Cell lines and cell culture

Human gastric cancer cell lines AGS (ATCC CRL-1739™ and gastric adenocarcinoma) and SNU-1 (CRL-5971™, gastric carcinoma) were purchased from ATCC. All cell lines were cultured according to the manufacturer’s instructions. Cells were harvested during logarithmic growth phase for the following experiments.

### Blood collection and serum preparation

Fasting blood (20 ml) was extracted from each participant in the morning of the day after admission. Blood samples were kept at room temperature for 2 h, followed by centrifugation at 2000 rpm for 15 min to collect serum samples.

### Real-time quantitative PCR

TRIzol reagent (Invitrogen, U.S.A.) was used to extract total RNA from tumor tissues, adjacent healthy tissues, and *in vitro* cultured cells. Using total RNA as template, cDNA was synthesized through reverse transcription using SuperScript III Reverse Transcriptase system (Thermo Fisher Scientific, U.S.A.). Following primers were used in PCR: 5′-AGCCCTAGATGGCTTTGTGA-3′ (forward) and 5′-TATCGAGGCTGTGTCGACTG-3′ (reverse) for HIF-1α; 5′-GACCTCTATGCCAACACAGT-3′ (forward), and 5′-AGTACTTGCGCTCAGGAGGA-3′ (reverse) for β-actin. PCR conditions were: 95°C for 38 s, followed by 40 cycles at 95°C for 12 s, and 58°C for 30 s. *C*_T_ values were processed using 2^−ΔΔ*C*^_T_ method, and relative expression level of HIF-1α was normalized to endogenous control β-actin.

### Establishment of HIF-1α siRNA silencing cell lines

HIF-1α siRNA silencing cell lines were constructed using commercial HIF-1α siRNA (catalog number AM16708, Thermo Fisher Scientific, U.S.A.). siRNAs. *Silencer*™ Select Negative Control No. 1 siRNA (catalog number 4390843, Thermo Fisher Scientific, U.S.A.) was included as an endogenous control. Cells were cultured overnight to reach 80–90% confluence before transfection. Transfection was performed by incubating with Lipofectamine 2000 reagent (11668-019, Invitrogen, Carlsbad, U.S.A.) for 4 h, and all other operations were performed according to the instructions.

### ELISA

Serum levels of HIF-1α were measured by ELISA using a kit provided by Thermo Fisher Scientific (U.S.A.). Absorbance at 450 nm was measured. Each measurement was performed for three times, and serum levels of HIF-1α were determined according to a standard curve that generated for each set of assayed samples. Assay sensitivity was 1 pg/ml.

### Cell proliferation assay

Cell counting kit (CCK-8, Sigma–Aldrich, U.S.A.) was used to evaluate cell proliferation ability of each cell line under different treatments according to the instructions. Briefly, 5 × 10^3^ cells in 100 μl were transferred to each well of 96-well plates. Cells were cultured at 37°C and CCK-8 solution (10 μl) was added into each well 24, 48, 72, and 96 h later. After incubation at 37°C for another 4 h, OD values at 450 nm were measured using a microplate reader (Bio-Rad, U.S.A.).

### Cell migration and invasion assays

Cell migration ability was detected by Transwell cell migration assay (BD Biosciences, U.S.A.). In brief, the upper chamber was filled with serum-free RPMI-1640 medium containing 5 ×10^4^ cells, and the lower chamber was filled with RPMI-1640 medium (Thermo Fisher Scientific, U.S.A.) containing 20% FBS (Sigma–Aldrich, U.S.A.). After incubation for 24 h, membranes were collected and stained with 0.5% Crystal Violet (Sigma–Aldrich, U.S.A.) for 20 min. Stained cells were counted under an optical microscope (Olympus, Japan). Invasion assay was performed using the same way, but the upper chamber was precoated with Matrigel (356234, Millipore, U.S.A.) before use.

### Western blot

Cell lysis solutions (Thermo Fisher Scientific, U.S.A.) were used to extract total RNA, and protein samples were quantitated by BCA assay. After that, 10% SDS/PAGE gel electrophoresis was carried out using 20 µg protein from each sample, followed by gel transfer to PVDF membranes. After blocking with 5% skimmed milk at room temperature for 1 h, PDVF membranes were washed and incubated with primary antibodies including rabbit anti-AKT antibody (1:2000, ab126811, Abcam), rabbit anti-phosphor-AKT antibody (p-T308, 1:2000, ab38449, Abcam), rabbit anti-VEGF antibody (1:2000, ab46154, Abcam), and anti-GAPDH antibody (1:1000, ab9485, Abcam) overnight at 4°C. The next day, membranes were washed with TBST and incubated with anti-rabbit IgG-HRP secondary antibody (1:1000, MBS435036, MyBioSource) at room temperature for 2.5 h. Then membranes were washed again with TBST, and signals were detected by ECL (Sigma–Aldrich, U.S.A.) method. Relative expression level of each protein was normalized to endogenous control GAPDH using ImageJ.

### Statistical analysis

Statistical analyses were performed using SPSS19.0 (SPSS Inc., U.S.A.). Measurement data were recorded by (x ± s), and comparisons between two groups were performed by *t* test. Count data were expressed as rate and percentage, and comparisons were performed using Chi square test. *P*<0.05 was considered to be statistically significant.

### Ethics approval and consent to participate

The present study was approved by the Ethics Committee of Shanxi Provincial People’s Hospital. All participants signed informed consents.

## Results

### Expression of *HIF-1α* mRNA was increased in tumor tissues compared with adjacent healthy tissues

**Figure 1:**
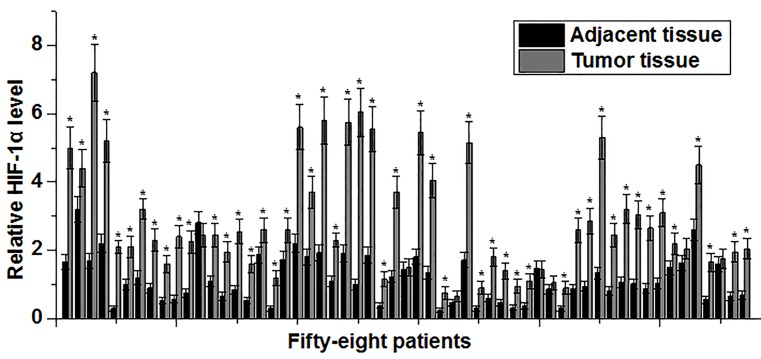
Expression of *HIF-1α* mRNA in tumor tissues and adjacent healthy tissues Expression level of *HIF-1α* mRNA was significantly higher in tumor tissues than in adjacent healthy tissues in most patients. *Compared with tumor tissues, *P*<0.05.

Expression of *HIF-1α* mRNA in tumor tissues and adjacent healthy tissues of 58 gastric cancer patients was detected by qRT-PCR. As shown in Figure 1, expression level of *HIF-1α* mRNA was significantly increased in tumor tissues compared with adjacent healthy tissues in 52 out of 58 patients. Those data suggest that HIF-1α overexpression may be involved in the pathogenesis of gastric cancer.

### Comparison of serum levels of HIF-1α protein between gastric cancer patients and normal healthy people and the diagnostic value

**Figure 2:**
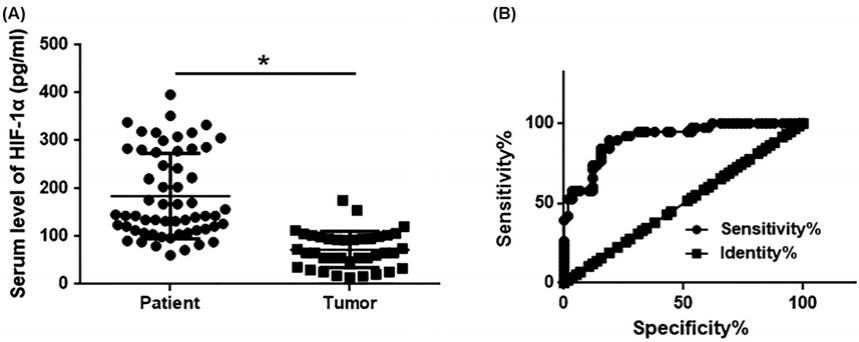
Serum levels of HIF-1α protein in gastric cancer patients and normal healthy people with the diagnostic value (**A**) Comparison of serum levels of HIF-1α protein between gastric cancer patients and normal healthy people. (**B**) ROC curve analysis of the diagnostic value of serum levels of HIF-1α protein for gastric cancer. Up-regulated serum levels of HIF-1α effectively distinguished gastric cancer patients from healthy controls. *, *P*<0.05.

ELISA was used to measure levels of HIF-1α protein in serum of gastric cancer patients and normal healthy people. As shown in Figure 2, serum levels of HIF-1α protein were significantly higher in gastric cancer patients than in normal healthy people. ROC curve analysis showed that the area under curve (AUC) of serum HIF-1α in the diagnosis of gastric cancer was 0.9068 with 95% confidence interval of 0.8482–0.9653 (*P*<0.001). Those results suggest that serum HIF-1α may serve as a promising diagnostic marker for gastric cancer.

### Correlations between serum level of HIF-1α with clinical features of gastric patients and its diagnostic value

**Figure 3:**
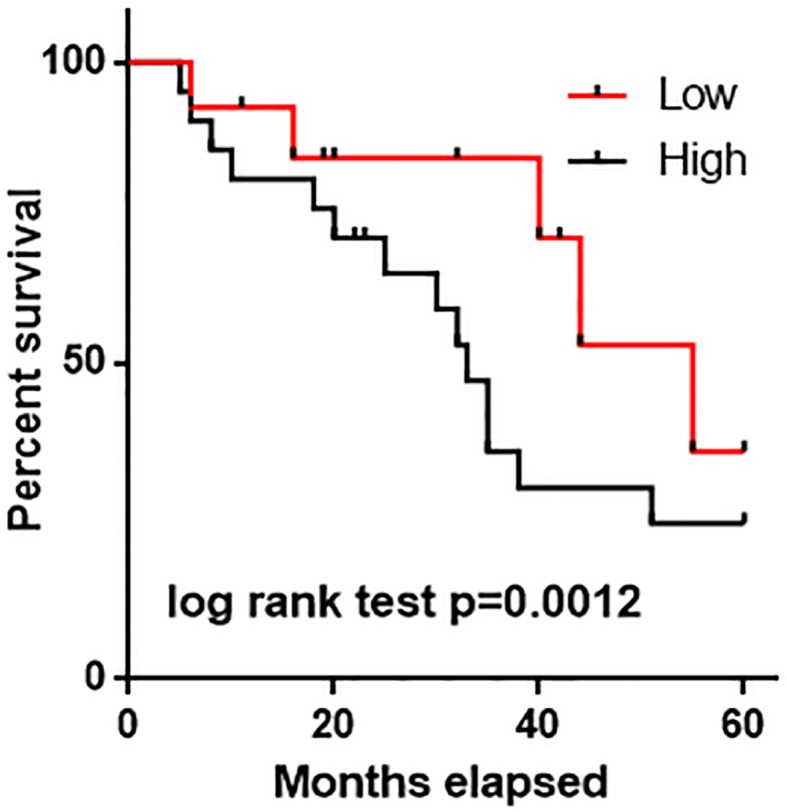
Comparison of survival curves of patients with high and low serum HIF-1α level Overall survival of gastric cancer patient with high expression level of HIF-1α was significantly shorter than that of patients with low expression level of HIF-1α.

As shown in Table 1, no significant differences in serum levels of HIF-1α were found between male and female patients (*P*>0.05). In addition, no significant differences in serum levels of HIF-1α were found between different age and habit groups (vegetarian, smoking, and drinking, *P*>0.05). However, serum levels of HIF-1α were significantly higher in patients with tumor diameter >5 cm than in patients with tumor diameter <5 cm (*P*<0.05). Besides that, serum levels of HIF-1α were also significantly higher in patients with tumor metastasis than in patients without metastasis (*P*<0.05). Patients were divided into high HIF-1α expression group (*n*=29) and low HIF-1α expression group (*n*=29) according to the median level of serum HIF-1α. Kaplan–Meier method was used to draw survival curves to evaluate the prognostic value of serum HIF-1α for gastric cancer. Log-rank test was used to compare survival curves. As shown in Figure 3, overall survival of gastric cancer patients with high expression level of HIF-1α was significantly shorter than that of patients with low expression level of HIF-1α (*P*<0.05). The results suggest that HIF-1α expression may serve as a prognostic marker for patients with gastric cancer.

**Table 1:** Correlations between serum levels of HIF-1α with clinical features of gastric patients

Items	Groups	Serum HIF-1α (pg/ml)	*P*-value
Gender	Male	264.26 ± 68.83	*P*>0.05
	Female	259.26 ± 77.45	
Age (years)	>40	271.26 ± 72.33	*P*>0.05
	<40	261.26 ± 77.12	
Vegetarian	Yes	271.26 ± 72.33	*P*>0.05
	No	261.26 ± 77.12	
Smoking	Yes	271.26 ± 72.33	*P*>0.05
	No	261.26 ± 77.12	
Drinking	Yes	271.26 ± 72.33	*P*>0.05
	No	261.26 ± 77.12	
Diameter (cm)	>5	330.26 ± 66.23	*P*>0.05
	<5	198.26 ± 45.12	
Metastasis	Yes	321.26 ± 55.45	*P*>0.05
	No	201.26 ± 44.43	

### Down-regulation of HIF-1α inhibited the proliferation, migration, and invasion of gastric cancer cells

**Figure 4:**
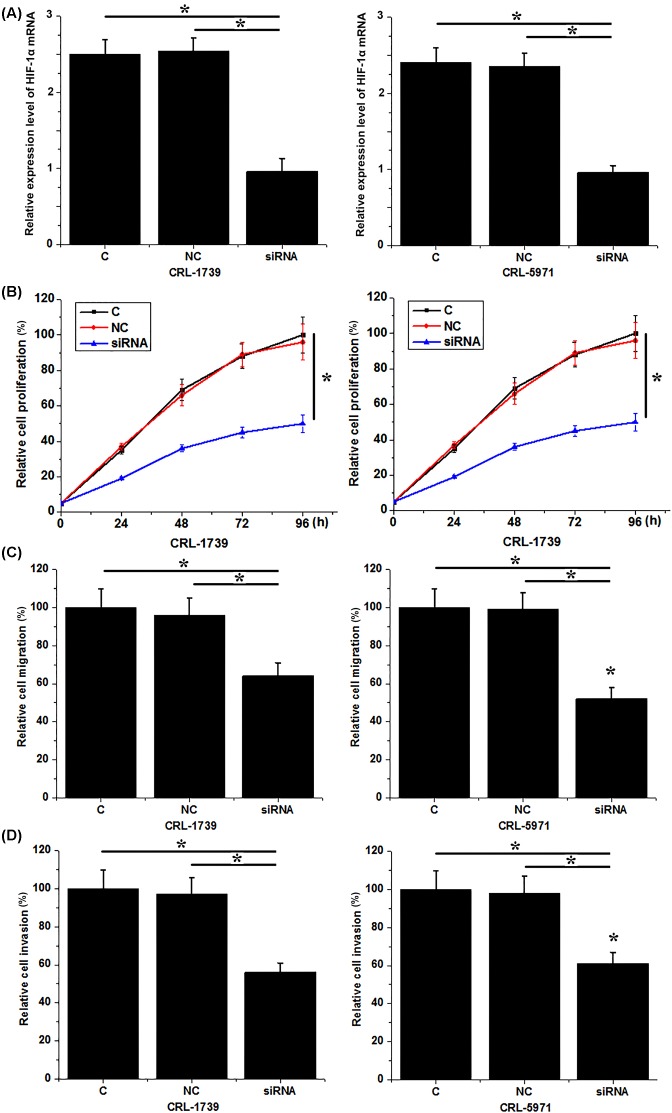
Down-regulation of HIF-1α inhibited proliferation, migration, and invasion of gastric cancer cells (**A**) Down-regulation of HIF-1α after siRNA transfection; (**B**) down-regulation of HIF-1α inhibited proliferation of gastric cancer cells; (**C**) down-regulation of HIF-1α inhibited migration of gastric cancer cells; and (**D**) down-regulation of HIF-1α inhibited invasion of gastric cancer cells. *P<0.05.

Compared with untransfected cells (control) and cells that transfected with control siRNA (negative control), expression level of *HIF-1α* mRNA was significantly reduced in cells that transfected with HIF-1α siRNA (Figure 4A), indicating the successfully established HIF-1α siRNA silencing cell lines. Compared with control cells, cell proliferation (Figure 4B), migration (Figure 4C), and invasion (Figure 4D) of both CRL-1739 and CRL-5971 were significantly inhibited after HIF-1α siRNA transfection. These results suggest that down-regulation of HIF-1α can inhibit proliferation, migration, and invasion of gastric cancer cells.

### Down-regulation of HIF-1α inhibited PI3K/AKT pathway and VEGF

**Figure 5:**
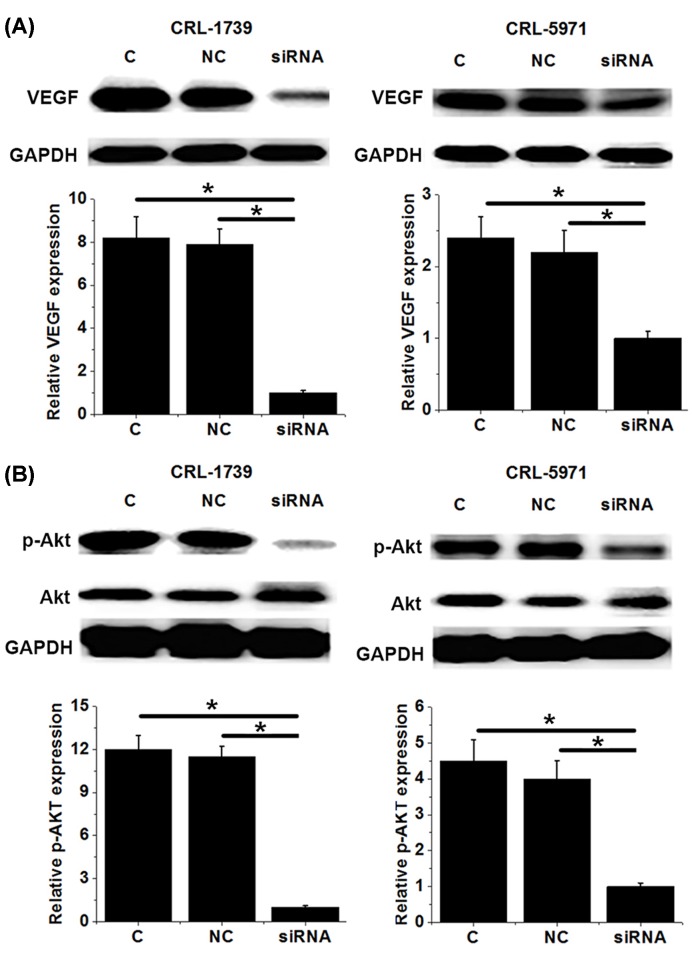
Down-regulation of HIF-1α inhibited PI3K/AKT pathway and VEGF This figure shows the effects of HIF-1α siRNA silencing on expression of VEGF (**A**) and phosphorylation and expression of AKT (**B**). In (A,B) the upper part is Western blot results and the lower part is the normalized expression. HIF-1α siRNA silencing inhibited VEGF expression and AKT phosphorylation. *, *P*<0.05.

Our data have shown that HIF-1α has important functions in the development and progression of gastric cancer. VEGF, as a key factor in angiogenesis, also plays pivotal roles in tumor growth and development. In the present study, HIF-1α siRNA silencing significantly reduced the expression level of VEGF in two gastric cancer cell lines (*P*<0.05, Figure 5A). In addition, HIF-1α siRNA silencing also significantly reduced the phosphorylation level of AKT (*P*<0.05), but showed no significant effects on its expression level (Figure 5B). These data suggest that HIF-1α may interact with VEGF and PI3K/AKT pathway to participate in the pathogenesis of gastric cancer.

### PI3K activator reduced the inhibitory effects of down-regulation of HIF-1α on proliferation, migration, and invasion of gastric cancer cells

**Figure 6:**
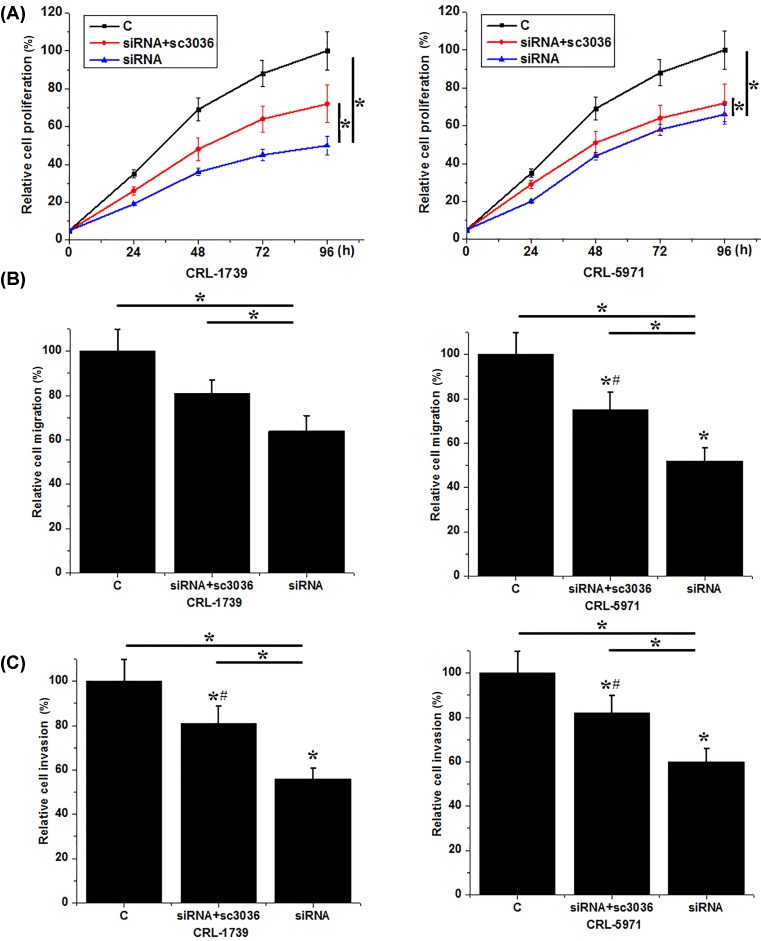
Effects of PI3K activator sc-3036 on proliferation, migration, and invasion of gastric cancer cells (**A**) Effects of PI3K activator sc-3036 on proliferation of gastric cancer cells; (**B**) effects of PI3K activator sc-3036 on migration of gastric cancer cells; (**C**) effects of PI3K activator sc-3036 on invasion of gastric cancer cells. Treatment with sc-3036 significantly reduced the inhibitory effects of HIF-1α siRNA silening on cell proliferation, migration, and invaison. *, *P*<0.05, compared with group C; #, *P*<0.05, compared with group siRNA.

PI3K activator sc-3036 (10 nm, Santa Cruz Biotechnology) was used to treat gastric cells with HIF-1α siRNA silencing. No significant changes were found in expression level of HIF-1α between gastric cancer cells with and without sc-3036 (data not shown) treatment, indicating that the activation of PI3K has no significant effects on expression of HIF-1α in gastric cancer cells. As shown in Figure 6, cell proliferation (Figure 6A), migration (Figure 6B), and invasion (Figure 6B) abilities of two gastric cancer cell lines with HIF-1α siRNA silencing were significantly increased after sc-3036 treatment, but were still significantly lower than those of control cells (untransfected cells). These data suggest that HIF-1α may serve as an upstream regulator of PI3K/AKT pathway to regulate the proliferation, migration, and invasion of gastric cancer cells.

## Discussion

HIF-1α is a subunit of a heterodimeric transcription factor hypoxia-inducible factor 1 [10]. Previous studies have shown that hypoxia and genetic alternations induce abnormal expression of HIF-1α, which is closely correlated with the development and progression of various types of malignancies [11]. In the study of breast cancer, Saponaro et al. [12] found that HIF-1α expression was up-regulated in breast cancer tissues compared with adjacent healthy tissues, and the increased expression level of HIF-1α contributed to the formation of new tumor tissues. In another study, up-regulation of HIF-1α expression was also observed in patients with ovarian cancer, and HIF-1α overexpression in ovarian tumor tissues not only accelerated the development and progression of cancer, but also induced the formation of drug resistance, which in turn led to poor treatment outcomes [13]. Up-regulated expression of HIF-1α has also been observed in gastric cancer. HIF-1α overexpression in gastric tumor tissues triggers abnormal expression of a gene network to promote the progression of cancer [14]. In the present study, expression level of HIF-1α was found to be significantly higher in tumor tissues than in adjacent healthy tissues in 52 out of 58 gastric cancer patients. In addition, serum levels of HIF-1α were also significantly higher in gastric cancer patients than in normal healthy people. Our findings and previous studies show that up-regulation of HIF-1α is highly likely to be involved in the pathogenesis of gastric cancer.

Development of diseases is usually accompanied by changes of certain substances in blood, and detection of the changes in those substances may provide references for the diagnosis of diseases. A previous study showed that increased expression level of HIF-1α usually predicts poor treatment outcomes of chemoendocrine therapy and low disease-free survival rate in patients with primary breast cancer [15]. In the study of cervical cancer, HIF-1α overexpression was also proved to predict early-stage tumor invasion and unfavorable prognosis [16]. In our study, serum level of HIF-1α was not affected by age and gender. However, serum levels of HIF-1α were significantly higher in patients with tumor diameter >5 cm than in patients with tumor diameter <5 cm. Besides that, serum levels of HIF-1α were also significantly higher in patients with tumor metastasis than in patients without metastasis. Individuals’ living habits such as smoking [17], drinking [18], and being vegetarian [19] can regulate the expression of certain genes in human body through methylation and other pathways. Therefore, individuals’ living habits may also affect the accuracy of certain biomarkers in the diagnosis of diseases. In our study, no significant effects of smoking, drinking, and being vegetarian on serum HIF-1α were observed. All these data indicate that serum HIF-1α is a sensitive and reliable prognostic and diagnostic marker for gastric cancer.

HIF-1α participates in the pathogenesis of different types of malignancies by regulating the proliferation, migration, and invasion of cancer cells. In the study of bladder cancer, HIF-1α overexpression promoted proliferation, migration, and invasion of cancer cells by targetting long noncoding RNA urothelial carcinoma associated 1 [20]. In contrast, HIF-1α knockdown in glioma cells inhibited cell migration *in vitro* and cell invasion *in vivo* [21]. In our study, HIF-1α siRNA silencing significantly reduced the proliferation, migration, and invasion abilities of cancer cells. HIF-1α was reported to regulate the proliferation and invasion of non-small cell lung cancer cells by interacting with PI3K/AKT pathway [22]. In our study, HIF-1α siRNA silencing showed no significant effects on expression of AKT, but significantly reduced the phosphorylation level of AKT. In addition, treatment with PI3K activator sc3036 significantly reduced the inhibitory effects of HIF-1α siRNA silencing on proliferation, migration, and invasion of gastric cancer cells. HIF-1α in some cases may achieve its biological functions by regulating the expression of VEGF [23]. In our study, HIF-1α siRNA also significantly reduced the expression level of VEGF. These data suggest that down-regulation of HIF-1α can inhibit the proliferation, migration, and invasion of gastric cancer by inhibiting PI3K/AKT pathway and VEGF expression.

## Conclusion

Expression of HIF-1α was significantly higher in tumor tissues than in adjacent healthy tissues in most gastric cancer patients. Serum levels of HIF-1α were also higher in gastric cancer patients than in normal healthy people. Serum HIF-1α showed high diagnosic and prognostic values for gastric cancer. HIF-1α siRNA silencing inhibited the proliferation, migration, and invasion of gastric cancer cells, while PI3K activator sc3036 treatment reduced those inhibitory effects. So we conclude that down-regulation of HIF-1α can possibly inhibit the proliferation, migration, and invasion of gastric cancer by inhibiting PI3K/AKT pathway and VEGF expression. The present study is limited by the small sample size. Future studies with bigger sample size are needed to further confirm the conclusions.

## Abbreviations

CCK-8: cell counting kit
HIF-1α: hypoxia-inducible factor 1α
HRP: horseradish peroxidase
TBST: tris-buffered saline
